# Microbial oil production by *Yarrowia lipolytica* strains using glycerol as carbon source

**DOI:** 10.1007/s42770-026-02054-z

**Published:** 2026-08-18

**Authors:** Elisa Duarte Guerra Coelho, Fernanda Palladino, Elisa A. A. Teixeira, Fernanda L. C. Oliveira, Vânya M. D. Pasa, Luiz H. Rosa, Carlos A. Rosa

**Affiliations:** 1 Departamento de Microbiologia, ICB, Universidade Federal de Minas Gerais, Belo Horizonte, C. P. 486, 31270-901 MG Brazil; 2 Departamento de Química, Biotecnologia e Engenharia de Bioprocessos, Universidade Federal de São João del-Rei, Ouro Branco, 36307-352 MG Brazil; 3 Departamento de Química, ICEX, Universidade Federal de Minas Gerais, Belo Horizonte, 21270- 901 MG Brazil

**Keywords:** Microbial oil, Oleaginous yeast, *Yarrowia lipolytica*, Glycerol, Biodiesel

## Abstract

Yeasts can produce microbial oil from hydrophobic substrates such as glycerol, a byproduct of the biodiesel industry that is often undervalued in the market. *Yarrowia lipolytica* is capable of degrading glycerol and is considered a model organism for studying lipid accumulation. Different yeast strains and culture conditions can result in varying lipid yields with distinct fatty acid profiles. Thus, in this study, five strains of *Y. lipolytica* were tested for microbial oil production using pure glycerol at temperatures of 15 °C and 25 °C. None of the isolates were considered oleaginous at 15 °C. However, at 25 °C, the isolates UFMG-CM-Y6114 (from cheese), UFMG-CM-Y4187 (from beach sand), and UFMG-CM-Y6189 (from Antarctica) produced more than 20% lipid content (29.30%, 26.95%, and 34.38%, respectively). No significant differences in lipid content were observed between the UFMG-CM-Y6114 and UFMG-CM-Y4187 strains. The cheese- and Antarctica-derived strains were further tested for their ability to accumulate oil when grown on crude glycerol. Among them, UFMG-CM-Y6189 showed the best performance, producing 9.40 g·L⁻¹ of biomass, 1.90 g·L⁻¹ of lipid yield, and 21.56% lipid content, while consuming 61.3 g·L⁻¹ of glycerol. The fatty acid profile of these lipids was predominantly monounsaturated (59.5%), with oleic acid (49.4%) as the main component. This, combined with a low polyunsaturated fatty acid content (5.2%), makes these lipids particularly suitable for biodiesel production. Therefore, the lipids from the *Y. lipolytica* strain UFMG-CM-Y6189 represent a promising raw material for biodiesel production from glycerol.

## Introduction

 Given the increasing global energy demand, the depletion of fossil fuel reserves, and the environmental impacts associated with their use, the search for renewable and sustainable energy sources—such as methane, biodiesel, bioethanol, and biohydrogen—has intensified [1–4]. Biodiesel stands out as a promising alternative due to its biodegradability, low toxicity, compatibility with existing engines, and reduced greenhouse gas emissions [5, 6]. Biodiesel can be categorized into three generations based on the raw materials used: the first generation is produced from edible vegetable oils; the second from non-edible oils and waste products; and the third from oleaginous microorganisms (such as microalgae, bacteria, and yeasts) that synthesize lipids—known as single-cell oils (SCO)—with fatty acid profiles similar to vegetable oils [7–9]. These microbial lipids are produced by oleaginous microorganisms through the conversion of carbon dioxide (CO₂), sugars, organic acids, hydrophobic substrates, and other carbon sources into intracellularly stored oils [5, 10, 11]. Many microbial species yield oils with fatty acid compositions comparable to those of vegetable oils used in first-generation biodiesel, making yeast-derived lipids suitable for third-generation biodiesel production.

In recent years, microbial lipids from oleaginous microorganisms have attracted considerable attention as potential biodiesel feedstocks due to their chemical similarity to vegetable oils, rapid growth rates, and ability to utilize a wide range of carbon sources [8, 12–14]. Several studies have investigated algal strains for biodiesel production. For example, *Chlorella protothecoides* UTEX 256 can accumulate up to 62% lipid content, while *Schizochytrium limacinum* SR21 can reach 71%, both using crude glycerol as a substrate [15–18]. However, microalgae-based biodiesel production faces significant challenges, including the requirement for large cultivation areas, vulnerability to climatic fluctuations and microbial contamination, and high water consumption for growth and processing [10, 19]. Furthermore, some microalgae produce oils with low monounsaturated fatty acid (MUFA) content and high polyunsaturated fatty acid (PUFA) content, which are undesirable for biodiesel production [20, 21].

Bacteria, by contrast, are easier to cultivate and exhibit faster growth rates than microalgae; however, they generally accumulate lower lipid contents. For example, *Acinetobacter calcoaceticus* stores 27–38% of its dry weight as lipids, whereas *Rhodococcus opacus* accumulates around 38% [18]. Given these limitations, oleaginous yeasts have emerged as leading candidates for third-generation biodiesel production. These microorganisms can be cultivated in bioreactors under controlled conditions, grow rapidly, and accumulate up to 80% of their dry biomass as lipids. Moreover, they can be genetically engineered to enhance oil yields and, as heterotrophic organisms, are capable of metabolizing diverse carbon sources, including industrial and agricultural by-products [10].

Oleaginous yeast species belong to the phyla *Ascomycota* (e.g., *Candida tropicalis*, *Cyberlindnera jadinii*, *Geotrichum fermentans*, *Kurtzmaniella cleridarum*, *Lipomyces tetrasporus*, *Metschnikowia pulcherrima*, *Myxozyma melibiosi*, *Torulaspora delbrueckii*, and *Yarrowia lipolytica*) and *Basidiomycota* (e.g., *Cryptococcus terricola*, *Leucosporidiella creatinivora*, *Moniliella spathulata*, *Papiliotrema laurentii*, *Pseudozyma aphidis*, *Rhodotorula toruloides*, *R. mucilaginosa*, *R. glutinis*, and *Trichosporon oleaginosus*) [8, 10, 22–25]. Among these, the literature highlights *Yarrowia lipolytica* as a particularly promising species for microbial lipid production using unrefined industrial by-products [24, 25].

Consequently, numerous studies have focused on identifying novel species and strains of microorganisms capable of producing high lipid concentrations from low-cost substrates, thereby improving economic feasibility. In parallel, research efforts have been directed toward developing efficient lipid extraction methodologies that minimize solvent use. Thus, the discovery of new lipid-accumulating yeast strains remains essential for advancing biodiesel production, ultimately enhancing the economic viability of this biotechnological process. This study underscores the potential of yeasts, particularly *Yarrowia lipolytica*, to convert glycerol—a low-cost industrial by-product—into microbial oil. The findings demonstrate the feasibility of employing native Brazilian yeast strains to produce oil with a fatty acid profile well-suited for biodiesel, characterized by high oleic acid content and enhanced oxidative stability. The aim of this study was to test different strains of *Yarrowia lipolytica* for lipid production using pure glycerol and crude glycerol used obtained from a biodiesel industry.

## Materials and methods

### Yeast strain, inoculum preparation and fermentation conditions

Five strains of *Y. lipolytica* were selected to evaluate microorganisms isolated from different substrates, including beach sand from Rio de Janeiro and Paraná, cheese, and lichens from Antarctica (Table 1). These isolates are preserved at − 80 °C in the Collection of Microorganisms and Cells of the Federal University of Minas Gerais.

**Table 1:** Average values of biomass, lipid yield, lipid content, glycerol consumption and (Y_L/S_) lipid yield per substrate in cultivation at 15 °C and 25 °C for *Yarrowia lipolytica* strains in 10 and 12 days cultured in pure glycerol

Yeast strain	Temperature	Days	Biomass in dry weight (g.L^− 1^)	Lipid (g.L^− 1^)	Lipid content (%)	Glycerol consumption (g.L^− 1^)	Y_L/S*_ (g.g^− 1^)
UFMG-CM-Y6114 (Cheese)	15	10	6.64 ± 0.07	0.65 ± 0.09	9.83 ± 1.27	38.67 ± 4.83	0.02
		12	7.45 ± 0.17	1.25 ± 0.00	16.79 ± 0.39	47.81 ± 3.47	0.03
	25	10	6.50 ± 0.27	1.64 ± 0.09	25.21 ± 0.28	42.26 ± 2.59	0.04
		12	6.78 ± 0.29	1.98 ± 0.24	29.30 ± 4.12	48.52 ± 2.00	0.04
UFMG-CM-Y327 (Cheese)	15	10	6.37 ± 1.54	0.55 ± 0.11	8.72 ± 1.12	47.17 ± 8.38	0.01
		12	8.48 ± 0.21	0.69 ± 0.03	8.18 ± 0.35	49.86 ± 8.27	0.01
	25	10	4.43 ± 0.17	0.71 ± 0.06	16.14 ± 1.79	48.66 ± 8.86	0.01
		12	6.37 ± 0.20	0.87 ± 0.14	13.58 ± 1.72	50.97 ± 2.29	0.02
UFMG-CM-Y6189 (Lichen - Antarctica)	15	10	6.55 ± 0.54	0.51 ± 0.10	7.81 ± 1.11	31.97 ± 12.77	0.02
		12	7.68 ± 0.35	1.21 ± 0.06	15.80 ± 1.47	34.76 ± 4.34	0.03
	25	10	7.58 ± 0.42	1.73 ± 0.12	22.88 ± 1.30	50.03 ± 2.03	0.03
		12	8.35 ± 0.30	2.87 ± 0.11	34.38 ± 2.15	60.68 ± 1.81	0.05
UFMG-CM-Y4187 (Beach - Paraná)	15	10	7.67 ± 0.14	0.90 ± 0.38	11.74 ± 5.11	37.27 ± 7.57	0.02
		12	8.55 ± 0.43	1.46 ± 0.30	17.05 ± 2.85	36.56 ± 0.85	0.04
	25	10	7.32 ± 0.11	1.58 ± 0.11	21.62 ± 1.20	43.35 ± 2.53	0.04
		12	7.63 ± 0.18	2.06 ± 0.37	26.95 ± 4.68	46.15 ± 3.21	0.04
UFMG-CM-Y4427 (Beach - Rio de Janeiro)	15	10	6.34 ± 1.81	0.45 ± 0.11	7.40 ± 2.93	41.24 ± 6.98	0.01
		12	9.45 ± 0.44	0.63 ± 0.09	6.72 ± 1.01	49.48 ± 6.12	0.01
	25	10	7.34 ± 0.28	0.82 ± 0.14	11.19 ± 2.02	50.05 ± 3.53	0.02
		12	8.23 ± 0.50	1.02 ± 0.07	12.42 ± 1.22	54.70 ± 4.10	0.02

* Y_L/S_: oil yield per substrate consumed

For the preparation of pre-inoculum, yeast strains were grown on medium YPD (glucose, 20 g·L^− 1^; peptone, 20 g·L^− 1^; yeast extract, 10 g·L^− 1^; and agar 20 g·L^− 1^) for 24 h at 28 °C. Each strain was grown in 125 and 250 mL Erlenmeyer flasks containing 35 and 75 mL of the glycerol, 100 g·L^− 1^; (NH_4_)_2_SO_4_, 1 g·L^− 1^; KH_2_PO_4_, 1 g·L^− 1^, MgCl_2_.6H_2_O, 0.5 g·L^− 1^ [26] and initial cell concentration of 0.5 g·L^− 1^ [27]. The 125 mL Erlenmeyer flasks were incubated at 15 °C and the 250 mL Erlenmeyer flasks at 25 °C, both in an orbital shaker at 150 rpm. All assays were conducted in triplicate, with samples collected every 48 h over 12 days. Each stage of the experiment (including cultivation in Erlenmeyer flasks, dry biomass determination, lipid extraction, and lipid quantification) was also performed in triplicate.

Based on the lipid production results using pure glycerol, the two best-performing strains were selected for an additional assay using crude glycerol as a carbon source. The crude glycerol was obtained from a biodiesel industry and had the following composition: 82.73% (w/w) glycerol, 6.3% sulphated ash, 5.15% NaCl, 3.3% chloride, 2.02% sodium, and 0.008% methanol [26]. It was used at a 10% concentration as the sole carbon source in the medium. For each analysis, dry biomass was measured, carbon source consumption was assessed, and lipid extraction and quantification were performed for each of the triplicates.

### Analytical methods

#### Cell concentration determination

Every two days, 10 mL of culture from 125 mL flasks and 30 mL from 250 mL flasks were transferred into pre-weighed 50 mL Falcon tubes. The tubes were centrifuged at 2,200 × g for 10 min, and 1 mL of the supernatant was collected for carbon consumption analysis. The resulting pellets were washed twice with 15 mL of distilled water. The tubes containing the pellets were then dried at 55 °C and weighed daily until a constant weight was achieved [26].

#### Determination of sugar concentration

Glycerol consumption was quantified using high-performance liquid chromatography (HPLC) with a Shimadzu system (Shimadzu, Japan). Culture samples were centrifuged at 2,600 × g for 15 min, and the resulting supernatants were diluted (1:10) and filtered through a Sep-Pak C18 (Millipore) filter. HPLC analysis was performed under the following conditions: Bio-Rad Aminex Supelco Analytical C-610 H column at 45 °C, Waters 410 refractive index detector, and an injection volume of 20 µL. The mobile phase consisted of 5 mM H₂SO₄ at a flow rate of 0.6 mL·min⁻¹ [27].

#### Lipid extraction

Every two days of cultivation, 10 mL of culture was transferred to a 50 mL Falcon tube and centrifuged at 2,200 × *g* for 10 min. The resulting pellet was washed twice with distilled water. The biomass was then resuspended in 15 mL of distilled water, and the cells were disrupted using a digital IKA Ultra-Turrax T18 homogenizer equipped with a dispersing element (S18N-19G) at 21,000 r.p.m. for two cycles of 2 min each, with a 1-minute interval between cycles. The disrupted cell suspension was transferred to a 100 mL Erlenmeyer flask, and 37.5 mL of a chloroform: methanol (2:1 v·v^− 1^) solution was added to extract the lipids [28]. The flasks were sealed and incubated in a shaker at 200 r.p.m. and 28 °C overnight. The liquid phase was then filtered through paper membranes, and the filtrate was centrifuged at 1,600 × g for 8 min to optimize phase separation. The upper phase, containing methanol and water, was carefully removed with a glass Pasteur pipette and discarded. The lower phase, containing chloroform and the extracted lipids, was transferred to a pre-weighed scintillation tube. The solvent was evaporated using a SpeedVac Concentrator (Savant SPD121P, Thermo Scientific, USA). After complete solvent removal, the scintillation tube was weighed again to determine the lipid yield. Using the dry biomass weight and the lipid weight, the lipid content percentage for each triplicate was calculated using Eq. 1 [26]:

$$\:\:\:\:\:\:\:\:\:\:\:\:\:\:\:\:\:\:\:\:\:\:\:\:\:\:\:\:\:\:\:\:\:\:\:\:\%\:lipid=\:\frac{Lipid\:yield\:\left({g.L}^{-1}\right)}{Dry\:weight\:\left({g.L}^{-1}\right)}\:x\:100$$Eq. 1.

#### Determination of fatty acids

Fatty acid detection was performed using gas chromatography. For pure glycerol samples, those collected on the 12th day of cultivation were analyzed. In tests using crude glycerol, samples were analyzed throughout the cultivation process. Only peaks that matched the standard reference peaks—representing 34 types of fatty acids—were considered. The area of each peak was used to determine its percentage relative to the total identified peaks.

For the characterization of fatty acids, cellular lipids were converted into fatty acid methyl esters (FAME) using the following procedure. Each oil sample was transferred to a vial, and 100 µL of a 1 mol·L⁻¹ potassium hydroxide solution was added. The vial was vortexed and incubated in a thermostatic bath at 65 °C for 20 min. After cooling, 400 µL of a 20% hydrochloric acid solution, 20 mg of sodium chloride, and 600 µL of ethyl acetate were added. The vial was vortexed again and left to stand for 5 min. Next, 300 µL of the upper organic phase was transferred to a new vial and evaporated under a nitrogen stream. Then, 100 µL of a 14% boron trifluoride solution in methanol was added. The vial was vortexed and incubated at 65 °C for 10 min. After cooling, 500 µL of HPLC-grade methanol and approximately 100 mg of anhydrous sodium sulphate were added. The solution was filtered using 0.2 μm pore filters.

FAME analysis was conducted using a gas chromatograph coupled with mass spectrometry (GCMS-QP5050A, Shimadzu, Japan) equipped with a DB-WAX capillary column (30 m · 0.25 mm · 0.25 μm). The detector and injector temperatures were set to 260 °C and 250 °C, respectively. The following temperature program was used for FAME separation: an initial temperature of 70 °C, increasing at a rate of 4 °C per minute until reaching a final temperature of 230 °C, which was maintained for 10 min. Helium was used as the carrier gas at a linear velocity of 28.1 cm·s⁻¹. Samples were injected at a volume of 1 µL with a split ratio of 1:50. FAME identification was performed using the Supelco 37 Component FAME Mix (10 mg·mL⁻¹, Sigma) as the standard, and quantification was carried out through area normalization. Each sample was analyzed over a 30-minute runtime [29].

#### *Statistical analyses*

All experiments were performed in triplicate. The means of the results were calculated and compared by analysis of variance (ANOVA) (p-value < 0.05).

## Results

### Screening for lipid-producing strains

**Fig. 1:**
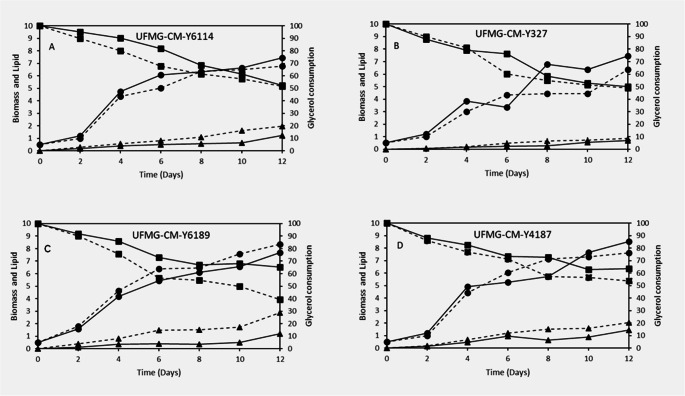
Formation of biomass (g.L^− 1^) (-●-), lipids (g.L^− 1^) (-▲-) and glycerol consumption (g.L^− 1^) (-■-) by different yeasts over 12 days at temperatures of 15 °C (─) and 25 °C (---)

Table 1; Fig. 1 present the results of assays conducted with pure glycerol at 15 °C and 25 °C, showing the mean values of triplicates and their respective standard deviations for the 10th and 12th days of cultivation. At 15 °C, none of the five *Y. lipolytica* strains produced more than 20% of their dry weight as lipids under the cultivation parameters and extraction methodology described in this study. Therefore, at this temperature, none of the strains can be classified as oleaginous. In contrast, at 25 °C, three of the five *Y. lipolytica* strains exceeded 20% lipid content on a dry weight basis, thereby qualifying as oleaginous yeasts under these conditions. Notably, these same three strains also displayed the highest lipid contents at 15 °C. Maximum lipid production for these strains occurred on the 12th day of cultivation, yielding: UFMG-CM-Y6114 (29.30% ± 4.12), UFMG-CM-Y4187 (26.95% ± 4.68), and UFMG-CM-Y6189 (34.38% ± 2.15). Furthermore, strains UFMG-CM-Y6189 and UFMG-CM-Y4187 reached 20% lipid content as early as the sixth day of cultivation. At 25 °C, the lipid yield and content of these three strains were more than twice those observed at 15 °C. Glycerol consumption was also higher at the elevated temperature. In UFMG-CM-Y6114 and UFMG-CM-Y4187, lipid accumulation increased as cell growth declined, indicating that oil synthesis predominantly occurred toward the end of the exponential growth phase. The progressive increase in glycerol consumption supports the hypothesis that the carbon source was being directed toward lipid biosynthesis, likely triggered by nitrogen depletion in the medium.

For strains UFMG-CM-Y327 and UFMG-CM-Y4427, biomass continued to increase while lipid yield remained constant (Fig. 1). This contrasts with the previously mentioned strains, where glycerol was primarily used for lipid accumulation rather than biomass formation. In strain UFMG-CM-Y6189, both lipid content and dry biomass increased alongside glycerol consumption, a trait advantageous for industrial processes as it reduces the lag before lipid production begins.

The Antarctic strain UFMG-CM-Y6189 demonstrated the highest biomass dry weight (8.35 ± 0.30 g·L⁻¹), lipid yield (2.87 ± 0.11 g·L⁻¹), and lipid content (34.38% ± 2.15). From the 10th to the 12th day, lipid yield increased by 65.9%. This strain also showed the highest glycerol consumption (60.68 ± 1.81 g·L⁻¹) and achieved the highest YL·S⁻¹ value in this study (0.05 g·g⁻¹).

Analysis of variance (ANOVA) (Table 2) revealed statistically significant differences among the yeast strains in terms of dry biomass production and lipid accumulation, as indicated by *p*-values below 0.05 and F values exceeding the critical F value. These results confirm that the observed variations are not attributable to random chance. In contrast, glycerol consumption showed no statistically significant differences between the strains, with *p*-values above 0.05 and F values below the critical threshold. This indicates that all strains utilized the substrate at comparable rates, suggesting a similar efficiency in glycerol metabolism across the tested isolates.

**Table 2:** Results of the Analysis of Variance (ANOVA) to evaluate the effect of the different yeasts on cell concentration, glycerol consumption, and lipid formation (*p* < 0.05)

	Temperature	*F*	*p-value*	*F-critical*
Biomass in dry weight (g.L^− 1^)	15 °C	5.491899	0.044946	5.192168
	25 °C	33.52991	0.000835	5.192168
Lipid (g.L^− 1^)	15 °C	6.575049	0.031623	5.192168
	25 °C	14.65924	0.005717	5.192168
Glycerol consumption (g.L^− 1^)	15 °C	1.993043	0.233943	5.192168
	25 °C	4.094248	0.077132	5.192168

Based on gas chromatography results, the average fatty acid profiles of the oils were calculated from duplicate samples on the 12th day of cultivation. The results are presented in Table 3. Fatty acids with proportions below 2.0% were grouped under “Others.” The Table 3 also includes the total content of saturated, monounsaturated (MUFA), and polyunsaturated (PUFA) fatty acids.

**Table 3:** Lipid profile (%) of fatty acids produced by the five strains of *Yarrowia lipolytica* at temperatures of 15 and 25 °C, with 12 days of growth in pure glycerol

Yeast strain	Temperature (ºC)	Palmitic acid (C16:0) (%)	Palmitoleic acid (C16:1) (%)	Stearic acid (C18:0) (%)	Oleic acid (C18:1n9) (%)	Linoleic acid (C18:2n6) (%)	Others (%)	Saturated acids (%)	MUFA* (%)	PUFA* (%)
UFMG-CM-Y6114 (Cheese)	15	8.74 ± 0.0	16.51 ± 0.10	5.36 ± 0.0	63.44 ± 0.85	3.60 ± 0.16	2.35	15.68	80.44	3.87
	25	14.17 ± 0.3	7.6 ± 0.30	11.36 ± 1.16	58.51 ± 2.23	3.94 ± 0.35	4.42	27.89	67.84	4.27
UFMG-CM-Y327 (Cheese)	15	8.63 ± 0.43	13.89 ± 0.14	3.80 ± 0.0	67.65 ± 1.17	4.39 ± 0.22	1.64	13.19	82.01	4.81
	25	15.55 ± 0.54	5.68 ± 0.56	15.41 ± 0.81	54.78 ± 0.88	5.75 ± 0.32	2.83	31.71	62.03	6.27
UFMG-CM-Y6189 (Lichen - Antarctica)	15	8.34 ± 0.05	16.16 ± 0.50	4.21 ± 0.13	61.17 ± 0.78	6.23 ± 0.79	3.89	14.30	78.75	6.94
	25	18.40 ± 0.58	13.00 ± 2.46	7.63 ± 1.69	52.64 ± 2.67	5.33 ± 0.92	2.70	27.14	67.10	5.76
UFMG-CM-Y4187 (Beach - Paraná)	15	7.06 ± 0.30	15.01 ± 0.30	5.34 ± 0.32	66.14 ± 1.55	2.78 ± 0.15	3.68	14.26	82.08	4.05
	25	14.53 ± 0.06	7.67 ± 0.43	13.57 ± 0.12	57.78 ± 1.37	3.38 ± 0.26	3.07	29.43	66.82	3.75
UFMG-CM-Y4427 (Beach - Rio de Janeiro)	15	12.14 ± 0.73	16.06 ± 1.59	5.60 ± 0.56	60.25 ± 2.14	5.31 ± 1.32	0.63	18.08	76.61	5.31
	25	16.02 ± 0.17	11.01 ± 0.91	7.31 ± 0.75	59.02 ± 0.6	3.80 ± 0.25	2.13	24.46	71.73	3.80

*The values refer to the averages of the duplicates. MUFA: Monounsaturated fatty acids; PUFA: Polyunsaturated fatty acids

The fatty acid profiles of the five yeast strains were quite similar within each temperature condition, both in terms of the types of fatty acids present and their relative proportions. However, variations were observed between the two cultivation temperatures. The predominant fatty acid produced by the isolates at both temperatures was oleic acid (C18:1). At 15 °C, oleic acid composition ranged from 61.2% to 67.6%, followed by palmitoleic acid (C16:1), which varied between 13.9% and 16.5%. Palmitic acid (C16:0) was the next most abundant, with values between 7.1% and 12.1%. Stearic (C18:0) and linoleic (C18:2) acids were present in smaller amounts, ranging from 3.8% to 5.6% and 2.8% to 6.2%, respectively.

At 25 °C, two distinct fatty acid profile patterns were observed. In isolates UFMG-CM-Y6114, UFMG-CM-Y327, and UFMG-CM-Y4187, oleic acid (C18:1) remained the predominant component, ranging from 54.78% to 58.51%, followed by palmitic acid (C16:0) (14.17% to 15.55%) and stearic acid (C18:0) (11.36% to 15.41%). Palmitoleic acid (C16:1) appeared next, varying between 5.68% and 7.67%, while linoleic acid (C18:2) was present in smaller amounts (3.38% to 5.75%). In isolates UFMG-CM-Y6189 and UFMG-CM-Y4427, oleic acid (C18:1) also predominated, ranging from 52.64% to 59.02%, followed by palmitic acid (C16:0), which varied between 16.02% and 18.4%. The third most abundant fatty acid was palmitoleic acid (C16:1), with values between 11% and 13%, followed by stearic acid (C18:0) (7.31% to 7.63%). Linoleic acid (C18:2) was present in smaller proportions, ranging from 3.80% to 5.33%.

Differences in the proportions of palmitic (C16:0), palmitoleic (C16:1), and stearic (C18:0) acids between the two cultivation temperatures led to variations in the relative levels of saturated fatty acids (SFA), monounsaturated fatty acids (MUFA), and polyunsaturated fatty acids (PUFA). At both temperatures, MUFAs (primarily C16:1 and C18:1) were the predominant class, followed by SFAs (C16:0 and C18:0), with PUFAs (C18:2) occurring in smaller proportions. The most notable difference was that at 15 °C, the proportion of SFAs ranged from 13.2% to 18.1%, which was lower than the range observed at 25 °C (24.5% to 31.7%). Conversely, MUFA levels were higher at 15 °C (76.6% to 80.4%) compared with 25 °C (62.0% to 71.7%). PUFA levels remained relatively constant, varying between 3.8% and 6.9% at both temperatures. Fatty acids categorized as “others” included trace amounts (≤ 2.0%) of SFAs and MUFAs such as C17:0, C17:1, C18:3, C20:0, C20:1, and C24:0, which were not consistently present across all samples. These minor components were included in the total SFA, MUFA, and PUFA values.

Tests using pure glycerol at 15 °C and 25 °C were conducted to select the best lipid-producing strains, which were subsequently tested with residual crude glycerol from the biodiesel industry. This screening step was necessary, as oil production was expected to be lower when using crude glycerol. Therefore, strains that did not produce significant amounts of oil from pure glycerol were excluded from further testing. At 15 °C, none of the five strains produced more than 20% lipid content. However, at 25 °C, three oleaginous strains were identified. Of these, two were selected for further testing with crude glycerol at 25 °C: the cheese-derived yeast UFMG-CM-Y6114 and the Antarctic yeast UFMG-CM-Y6189.

### Evaluation of growth, substrate consumption and quantification of lipids produced from crude glycerol

In the experiments using crude glycerol, the cheese-derived isolate UFMG-CM-Y6114 failed to reach 20% lipid content, achieving only 15.88% ± 0.54 on the 12th day. This represents approximately half of the lipid content produced from pure glycerol at the same temperature. Although the biomass dry weight was higher with crude glycerol (8.74 ± 0.46 g·L⁻¹), the lipid yield was significantly lower (1.39 ± 0.07 g·L⁻¹). A better result was obtained with the Antarctic isolate UFMG-CM-Y6189, which reached a lipid content of 21.56% ± 1.04 on the 10th day. Although this was lower than the 34.38% obtained by the same strain in pure glycerol, as expected, it still exceeded the lipid production achieved by other isolates (UFMG-CM-Y327 and UFMG-CM-Y4427) in pure glycerol, as well as that of the cheese-derived yeast tested in crude glycerol. The maximum lipid yield of 1.90 ± 0.12 g·L⁻¹ was reached after 10 days by the Antarctic isolate. While this yield was lower than the maximum obtained by the same yeast in pure glycerol, it remained close to that of UFMG-CM-Y6114 in pure glycerol, which had the second-highest yield. Crude glycerol was used at a concentration of 10% (w·v^− 1^). Since it contains other compounds in addition to glycerol, its initial concentration in the medium was determined by liquid chromatography, yielding 91.98 g·L^− 1^. Table 4 presents the average values from triplicate measurements of crude glycerol consumption and the oil yield per substrate consumed (YL·S^− 1^) for both strains.

**Table 4:** Crude glycerol consumption, oil yield per consumed substrate (Y_L/S_), lipid profile (%) of fatty acids produced by strains of *Yarrowia lipolytica* UFMG-CM-Y6114 and UFMG-CMY6189 at temperatures of 25 °C with 10 and 12 days of growth in crude glycerol

Yeast strain	Days	Glycerol consumption	Y_L/S_	Palmitic acid (C16:0) (%)	Palmitoleic acid (C16:1)	Stearic acid (C18:0)	Oleic acid (C18:1n9)	Linoleic acid (C18:2n6)	Others	Saturated acids	MUFA*	PUFA*
UFMG-CM-Y6114 (cheese)	10	52.48 ± 2.76	0.02	14.46 ± 0.05	5.91 ± 0.02	16.34 ± 0.06	54.82 ± 0.16	4.03 ± 0.01	4.45	33.03	61.94	5.04
	12	60.00 ± 3.08	0.02	15.06 ± 0.03	5.67 ± 0.41	17.52 ± 0.01	54.10 ± 1.08	3.82 ± 0.04	3.83	34.15	61.59	4.26
UFMG-CM-Y6189 (lichen - Antarctica)	10	56.33 ± 1.11	0.03	21.98 ± 0.26	9.31 ± 0.36	11.49 ± 0.49	48.66 ± 0.66	4.61 ± 0.25	3.95	34.83	59.89	5.27
	12	61.31 ± 1.38	0.03	22.44 ± 0.08	9.18 ± 0.85	11.22 ± 0.45	50.10 ± 0.25	5.36 ± 0.05	1.70	33.62	60.81	5.57

*The values refer to the averages of the duplicates. MUFA: Monounsaturated fatty acids; PUFA: Polyunsaturated fatty acids

Glycerol consumption over time was very similar for both strains. The cheese-derived isolate consumed a higher concentration of crude glycerol (60.0 ± 3.08 g·L⁻¹) compared to pure glycerol (48.52 ± 2.0 g·L⁻¹), whereas the Antarctic isolate consumed approximately 60 g·L⁻¹ in both substrates. However, considering the percentage of glycerol consumed relative to the initial concentration in the medium, UFMG-CM-Y6114 utilized approximately 65.2% of the crude glycerol, while UFMG-CM-Y6189 consumed about 66.6%. The YL/S value for both yeasts was lower in crude glycerol, reaching 0.03 g·g⁻¹.

Table 4 presents the percentage composition of fatty acids, along with their classification into saturated fatty acids (SFA), monounsaturated fatty acids (MUFA), and polyunsaturated fatty acids (PUFA) produced from crude glycerol. In the cheese-derived isolate UFMG-CM-Y6114, the predominant fatty acid was oleic acid (C18:1) at 54.10%, followed by stearic acid (C18:0) at 17.52% and palmitic acid (C16:0) at 15.06%. In smaller amounts, palmitoleic acid (C16:1) was present at 5.67%, and linoleic acid (C18:2) at approximately 3.82%.

In the Antarctic isolate UFMG-CM-Y6189, oleic acid (C18:1) was also the most abundant fatty acid, comprising 50.10% of the total. Unlike UFMG-CM-Y6114, the second most predominant fatty acid was palmitic acid (C16:0) at 22.44%, followed by stearic acid (C18:0) at 11.22%. In smaller amounts, palmitoleic acid (C16:1) was present at 9.18%, and linoleic acid (C18:2) at 5.36%. The overall proportions of SFA, MUFA, and PUFA were relatively similar in both isolates. In UFMG-CM-Y6114, the content of saturated, monounsaturated, and polyunsaturated fatty acids was 40.58%, 61.59%, and 4.26%, respectively. In UFMG-CM-Y6189, the SFA, MUFA, and PUFA contents were 41.10%, 60.81%, and 5.57%, respectively.

## Discussion

For microbial oils to compete with vegetable oils in the biodiesel market, production costs must be reduced. The choice of raw material used as the microbial substrate plays a decisive role in these costs. Although purified glycerol offers optimal conditions for microbial growth and lipid accumulation, its industrial application is economically unfeasible due to the high purification costs. Consequently, crude glycerol—a by-product of the biodiesel industry—emerges as a more cost-effective alternative [14, 30, 31]. The initial tests at 15 °C and 25 °C using pure glycerol aimed to identify strains with the highest lipid-producing potential, which were subsequently evaluated using crude glycerol as the carbon source. Because crude glycerol contains impurities and potential inhibitory compounds, lipid production was expected to be lower compared with that obtained from purified glycerol [31]. Under specific cultivation conditions, only certain *Y. lipolytica* strains are considered oleaginous [32, 33]. This was confirmed in the present study, where three of the five strains tested with pure glycerol (UFMG-CM-Y6189, UFMG-CM-Y6114, and UFMG-CM-Y4187) and one of the two strains tested with crude glycerol (UFMG-CM-Y6189) accumulated more than 20% lipid content under the applied cultivation and extraction conditions.

The ability of microorganisms to adapt to low-temperature environments, such as Antarctica, is linked to the modulation of cell membrane fluidity through shifts in fatty acid composition from saturated to unsaturated forms [34, 35]. Thus, the high lipid content of the UFMG-CM-Y6189 strain—isolated from Antarctic lichen—may represent an adaptation to maintain membrane integrity, serving both structural and protective functions. In addition to maintaining membrane stability, intracellular lipid bodies act as energy reserves, a crucial advantage in nutrient-scarce environments like Antarctica [36].

In pure glycerol cultures at both temperatures, strains UFMG-CM-Y327 and UFMG-CM-Y4427 exhibited continued biomass accumulation without significant lipid production. In certain species, strains, or cultivation setups, the substrate is primarily directed toward biomass formation, with only a small fraction converted into lipids or by-products such as citric acid. Optimal lipid production occurs when both carbon and nitrogen consumption support biomass growth while promoting lipid accumulation—an outcome achieved by strains UFMG-CM-Y6114, UFMG-CM-Y6189, and UFMG-CM-Y4187 in pure glycerol at 25 °C.

For all isolates except UFMG-CM-Y6189, biomass values were higher at 15 °C than at 25 °C in pure glycerol. The reverse trend was observed for lipid yield, which was consistently higher at 25 °C. *Yarrowia lipolytica* can grow across a wide temperature range. Enshaeieh et al. [12] investigated the effects of different cultivation parameters—including temperatures of 15, 25, 35, and 45 °C—on lipid production by *Candida* sp. and *Y. lipolytica* using glucose as the carbon source. In their study, lipid production peaked at 25 °C in both yeasts, with *Y. lipolytica* achieving a maximum lipid content of 34.15%, while the lowest yield occurred at 35 °C. Similarly, in the present work, strains UFMG-CM-Y6189, UFMG-CM-Y6114, and UFMG-CM-Y4187 achieved the highest lipid contents at both 15 °C and 25 °C, but only surpassed the 20% lipid threshold in pure glycerol at 25 °C. Based on these results, two strains were selected for testing in crude glycerol at 25 °C: the Antarctic isolate UFMG-CM-Y6189, which displayed the best performance, and the cheese isolate UFMG-CM-Y6114.

Residual crude glycerol from biodiesel production—a by-product of the transesterification reaction—has a variable composition, containing nutrients such as copper, magnesium, iron, zinc, potassium, sodium, chlorine, and calcium [37, 38], as well as peptides, proteins, and additional nitrogen sources [26, 39]. This compositional variability likely explains the higher biomass values observed in crude glycerol cultivation for both isolates (UFMG-CM-Y6189: 9.40 ± 0.11 g·L⁻¹; UFMG-CM-Y6114: 8.74 ± 0.46 g·L⁻¹) compared with pure glycerol (UFMG-CM-Y6189: 8.35 ± 0.30 g·L⁻¹; UFMG-CM-Y6114: 6.78 ± 0.29 g·L⁻¹). However, increased biomass production did not translate into proportional lipid accumulation. The reduced lipid concentration in crude glycerol is likely due to a lower C/N ratio, which favors cell division over lipid synthesis [14, 31, 40]. The presence of minerals and nutrients in crude glycerol stimulates biomass growth, but at the expense of lipid yield. According to Kuttiraja et al. [40], C/N ratios above 50 are optimal for lipid accumulation, as nitrogen limitation redirects metabolism toward storage lipid biosynthesis.

Similar trends have been reported in other studies. Uprety et al. [41] observed higher biomass production in *R. toruloides* ATCC 10,788 cultivated in crude glycerol compared to pure glycerol. Likewise, Cheirsilp et al. [42] reported a biomass yield of 9.83 ± 0.10 g·L⁻¹ and a maximum lipid yield of 3.07 ± 0.03 g·L⁻¹ in *C. tropicalis* X37 using a fed-batch fermentation process with crude glycerol. In the present study, replacing pure glycerol with crude glycerol led to a 37.3% decrease in lipid content for the Antarctic isolate UFMG-CM-Y6189, from 34.38% ± 2.15 to 21.56% ± 1.01. A similar reduction (45.8%) was observed in the cheese isolate UFMG-CM-Y6114, with lipid content dropping from 29.30% ± 4.12 to 15.88% ± 0.54. Comparable results were reported by Poli et al. [26], where the QU21 isolate (UFMG-CM-Y327) showed increased biomass growth in crude glycerol but maintained the same lipid yield, resulting in a 37.2% drop in lipid content relative to pure glycerol cultivation. Table 5 presents biomass, yield, and lipid content data from previous studies on lipid production by wild-type *Y. lipolytica* strains using crude glycerol under conditions comparable to those of the present work. Notably, the UFMG-CM-Y6189 strain achieved the highest lipid yield (g·L⁻¹) and lipid content (%), underscoring its potential for sustainable glycerol valorization through industrial by-product utilization.

**Table 5:** Comparison of the results presented by different articles, in tests in crude glycerol with wild *Yarrowia lipolytica* strains in flasks with temperatures between 25–28 °C

Strain Y. lipolytica	Biomass (g.L^− 1^)	Lipid (g.L^− 1^)	Lipid content (%)	References
UFMG-CM-6189	8.81	1.9	21.56	This manuscript
LFMB 19	6.2	0.51	6.8	Chatzifragkou et al. (2011).
QU21	6.7	1.27	18.9	Poli et al. (2014)
ATCC 20,460	6.5	0.75	11.6	Sestric et al. (2014).
A101	6.63	1.32	19.9	Dobrowolski et al. (2016).
347	9.4	0.43	4.6	Canonico et al. (2016)

## Conclusion

In experiments using pure glycerol at 25 °C, three of the five *Y. lipolytica* strains—UFMG-CM-Y6114, UFMG-CM-Y4187, and UFMG-CM-Y6189—accumulated more than 20% lipid content. Both lipid yield and content were higher at 25 °C than at 15 °C, underscoring the critical influence of temperature on yeast-mediated oil biosynthesis. The fatty acid profiles of the five strains showed no substantial variation at the same cultivation temperature with pure glycerol; however, temperature differences affected the relative proportions of key fatty acids. At 15 °C, monounsaturated fatty acid levels were higher, while saturated fatty acid levels were lower compared to 25 °C. Across all strains, oleic acid (C18:1) accounted for more than 50% of the total fatty acids, and polyunsaturated fatty acids remained below 7%, meeting industry quality standards for biodiesel. Among the two strains tested with residual crude glycerol from the biodiesel industry, *Y. lipolytica* UFMG-CM-Y6189 achieved the highest lipid yield and content, accumulating 21.56% lipids and producing 1.9 g·L⁻¹ of oil. The lipids from this strain averaged 59.5% monounsaturated fatty acids, with oleic acid representing over 49% of the total fatty acid composition—characteristics well aligned with the requirements for high-quality biodiesel. These findings position *Y. lipolytica* UFMG-CM-Y6189 lipids as a promising feedstock for biodiesel production from crude glycerol, contributing to the sustainable valorization of industrial by-products.

## Data Availability

The data sets generated during and/or analyzed during the current study are available in the Microbiology Department, Biological Sciences Institute, Minas Gerais Federal University repository, <. https://repositorio.ufmg.br/bitstream/1843/42435/1/Disserta%C3%A7%C3%A3o%20Mestrado%20Elisa%20Guerra.pdf.
